# Application of URANS Simulation and Experimental Validation of Axial Flow-Induced Vibrations on a Blunt-End Cantilever Rod for Nuclear Applications

**DOI:** 10.1007/s13369-024-09505-5

**Published:** 2024-08-29

**Authors:** Anas Muhamad Pauzi, Hector Iacovides, Andrea Cioncolini, Hao Li, Mostafa R. A. Nabawy

**Affiliations:** 1 School of Engineering, The University of Manchester, Manchester, M13 9PL UK; 2 Department of Mechanical Engineering (Robotics), Guangdong Technion - Israel Institute of Technology (GTIIT), 241 Daxue Road, Shantou, 515063 Guangdong China

**Keywords:** Fluid–structure interaction (FSI), Nuclear fuel rod, Fretting wear, Optical tracking, Particle image velocimetry (PIV)

## Abstract

Fretting wear caused by flow-induced vibration (FIV) is a leading cause of fuel failure in light water nuclear reactors. This study describes a numerical methodology, validated with dedicated experiments, for predicting flow-induced vibrations in cantilever rods exposed to axial water flow, a paradigmatic configuration informative for fuel rods in water-cooled nuclear reactor cores. Utilising strong two-way fluid–structure interaction (FSI) simulations with an efficient computational approach, the study focuses on two key aspects of self-excited FIV: the dominant vibration frequency and the amplitude of the vibration. Correctly reproducing the former depends on optimising the solid domain and FSI coupling, while the latter hinges on the fluid solver’s ability to accurately replicate unsteady flow behaviour, especially in areas of flow separation. Two unsteady Reynolds averaged Navier–Stokes turbulence models, both being high-Reynolds number versions, and several discretisation schemes for the convection transport are evaluated for their capacity to reproduce the correct unsteady flow behaviour. When the axial flow is directed from the free end to the fixed end of the rod, both the Eddy viscosity model k-$\omega$ SST and the Reynolds stress model by Launder, Reece, and Rodi reliably predicted the frequency and amplitude of vibrations for a Reynolds number range between 16.4k and 61.7k. When the flow direction is reversed, while vibration frequencies were accurately modelled, replicating precise unsteady flow behaviour proved more challenging. The study underscores the importance of properly resolving the flow in areas of flow separation to achieve accurate simulation of unsteady flow behaviour.

## Introduction

Flow-induced vibrations (FIVs) are of concern in water-cooled nuclear reactors because they can cause a cyclic relative movement between the fuel rods and the support grids inside the reactor core, which in turn can lead to grid-to-rod fretting wear and subsequent cladding perforation and fuel failure. To date, grid-to-rod fretting has been recognised as the leading cause of 70% of all fuel failures observed in light water nuclear reactors [1], including the European Pressurised Reactor (EPR) recently completed in Tianshan, China, which experienced an extended shutdown from 2021 to 2022 due to excessive FIV localised in the reactor core. The fact that even newly built nuclear power stations are affected by grid-to-rod fretting clearly points to an unsatisfactory fundamental physical understanding and prediction capability of FIV in nuclear reactor cores. Even though excessive vibrations in fuel rods can be mitigated a posteriori by increasing the mechanical resistance of the fuel bundle support structure [2], there is a clear need to develop numerical methodologies that can accurately predict the occurrence of FIV in nuclear fuel bundles. Once validated, such numerical methodologies can then be employed in the design phase to support the development of nuclear fuel bundles that are less susceptible to FIV and thereby more fatigue resistant and more durable. This has motivated extensive research in recent years on numerically predicting FIVs at conditions relevant to nuclear reactor cores.

The type of flows that nuclear fuel rods are exposed to inside water-cooled reactor cores are axial flows, where the cooling water flows axially along the fuel rods through the gaps present between adjacent fuel rods. With axial-FIV, i.e. FIV caused by axial flows, the main cause of structural vibration is turbulent buffeting: the pressure fluctuations caused by the turbulence in the flow produce variable lateral loads on the rods, which in turn can trigger and sustain structural vibration. Therefore, a key aspect in numerical methodologies for axial-FIV is the selection of a turbulence model capable of reproducing enough unsteadiness in the flow to trigger and sustain the structural vibration.

High-fidelity large Eddy simulation (LES) turbulence modelling has been successfully used in a number of studies to reproduce the flow unsteadiness [3–5], which has then been coupled with structural solvers to predict the subsequent structural vibration [6–8]. The significant computational cost of LES simulations has motivated further investigations with the more cost-effective unsteady Reynolds averaged Navier–Stokes (URANS) turbulence models, which have been employed in a number of recent studies [9–15]. While most URANS studies have been quite successful in capturing the frequency of the structural vibration, reproducing the amplitude of vibration has proved to be considerably more challenging. In fact, the frequency of vibration mostly depends on the mechanical properties of the structure [16, 17] and is only minimally affected by the turbulent flow. On the other hand, the amplitude of the structural vibration directly depends on the magnitude of the flow unsteadiness. Therefore, the amplitude of the structural vibration will be underpredicted, as it was the case of most URANS studies documented to date, unless the turbulence model employed reproduces adequate unsteadiness in the flow. Sometimes, fine-tuning and ad hoc modifications have been proposed in order to improve the prediction accuracy of URANS turbulence models for axial-FIV. For example, Kottapalli et al. (2019) [12] implemented a stochastic model that generated additional pressure and velocity fluctuations in the flow, so as to increase the flow unsteadiness and thereby the amplitude of the structural vibrations.

Recent work by Salachna et al. [14] demonstrated the effectiveness of the URANS high-Reynolds-number Reynolds stress transport model proposed by Launder, Reece, and Rodi (LRR) [18] in successfully reproducing both the amplitude and the frequency of axial-FIVs observed in experiments with a cantilever rod exposed to turbulent water flow at a Reynolds number of 16.4k [16]. This same methodology was then successfully extended to a higher Reynolds number of 35.1k [15]. This paper further develops the axial-FIV numerical methodology originally proposed by Salachna et al. (2023) [14] by expanding the range of Reynolds numbers investigated (up to 61.7k), by incorporating new high-resolution experimental data which have been generated specifically to support the further development of the numerical methodology and by assessing the implementation of the k-$\omega$ SST turbulence model in addition to the URANS LRR model previously employed.

## Benchmarking Experiment

**Fig. 1:**
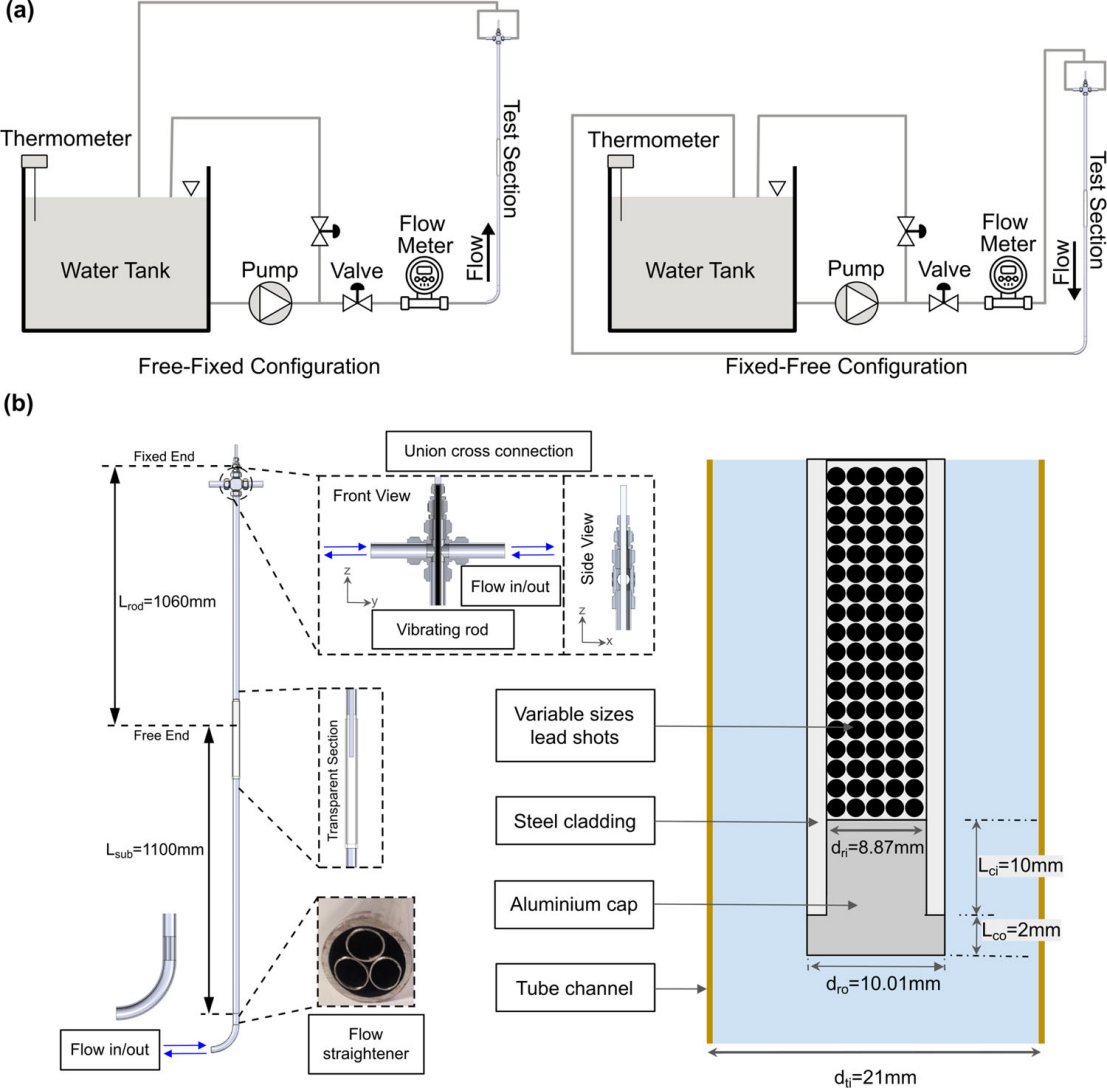
**a** Schematic of the experimental setup for the two configurations tested. **b** Schematic of the test piece section (left) and details of the cantilevered rod at the free end (right)

Dedicated axial-FIV experiments were conducted at the University of Manchester (UoM) to validate the numerical methods that will be presented in this paper. The experimental rig used for measurements is similar to the one presented in the study of Cioncolini et al. [17, 19]. As such and for brevity, only the main aspects of the setup will be reiterated here. The setup involves a single cantilever rod tested under two different flow configurations: a free–fixed configuration where water flows from the rod’s free end towards the rod’s fixed end, or a fixed–free configuration where the water flows from the rod’s fixed end towards the rod’s free end. Detailed schematics of the experimental setup, test section, and the cantilevered rod are provided in Fig. 1.

A flow straightener was positioned at the lower end of the confining tube hosting the rod. In the case of bottom-up flows (free–fixed configuration), the upstream flow passes through the straightener to eliminate any swirling and secondary flows, while achieving minimal pressure losses. The configuration of the straightener is shown in the insert inside Fig. 1b. At the uppermost part, the test piece is linked to two symmetrically routed pipes. This connection is achieved through a union cross (model: Swagelok SS-25M0-4), which also serves to secure the cantilever boundary condition of the vibrating rod. To prevent any potential impact on the rod dynamics, a symmetric two-pipe setup was employed instead of a conventional one-pipe arrangement, to eliminate any localised cross-flow near the fixed end of the rod. These pipes have the same size as the test section confining tube, with an inner diameter of $20.86 \pm 0.05$ mm and an outer diameter of $25.06 \pm 0.01$ mm.

In the free–fixed configuration, the flow enters the lower part of the test section and ascends vertically from the free end of the rod towards the fixed end, ultimately exiting the test section at the union cross. The distance between the top of the flow straightener and the free end of the vibrating rod is 1100 mm, which is equivalent to a flow recovery length of 52.7 times the diameter of the confining pipe. Therefore, the flow straightener and the flow recovery length adequately ensure that the flow reaches fully developed conditions before reaching the free end of the vibrating rod.

In the fixed–free flow configuration, the flow enters the test section from the top and flows in a downward vertical direction, starting at the top end where the rod is clamped and exiting the test section at the bottom. The axial flow along the vibrating rod occurs when the two incoming streams enter the union cross from both sides: this flow is influenced by the mixing and change in direction of the two streams, as they transit from the horizontal to the vertical direction. However, this flow is relatively less affected by disturbances in the two feeding pipes upstream. Therefore, flow straighteners were not fitted on the two feeding lines located at the top of the test section.

The vibrating rod, hosted in the confining tube, was made of a circular tube of AISI 316 stainless steel with the dimensions shown in Fig. 1b. The surface of the tube had a roughness of 2–3 $\mu$m. The hydraulic diameter of the annular gap between the confining tube and the vibrating rod was 10.85 mm. The rod had a *blunt-shaped* end-piece, as depicted in Fig. 1b. In order to mitigate any potential local mass effects caused by the end-piece on the dynamics of the rod, the end-piece was designed to be lightweight, ensuring that it does not significantly change the linear mass density of the vibrating rod. The vibrating rod was packed with lead shots, which had a density of 11.34 g $\hbox {cm}^{-3}$ and diameters ranging from 0.3 to 1.6 mm, allowing the replication of the loading of fuel pellets in nuclear fuel bundles. The rod’s mass density, taking into consideration both the rod material and the lead shot loading, was 588 g $\hbox {cm}^{-1}$.

As the water flows past the cantilevered rod, a particle image velocimetry (PIV) system was used to measure the flow fields at two locations: first, a few diameters beneath the cantilevered rod to ensure the flow is consistently a fully developed pipe flow (see later results in Sect. 4.2), and second, near the rod’s free end to capture the flow field, including flow separation and reattachment. The tests were conducted using a Dantec Dynamics PIV system, which includes a double cavity Nd:YLF laser capable of producing a maximum output energy of 15 mJ at a wavelength of 527 nm. The laser was connected to an articulated light arm with a beam-width-controlling optic and a diverging lens to spread the laser light in a vertical plane across the middle of the flow field. The laser sheet had a thickness of roughly 1 mm and was positioned at an angle to the flow direction.

The process of capturing the flow images on double frames involved utilising a high-speed camera (Phantom V310) operating at 200 fps, with a resolution of 1280 $\times$ 800 pixels, and employing a Nikon AF Micro 60 mm f/2.8D lens. The velocity field mapping was derived from the displacement of particles introduced into the flow, where hollow glass spherical particles with a nominal size of 10 $\mu$m were used. Following the addition of the particles, the rig was operated at its maximum flow rate for a few minutes to ensure thorough circulation and uniform dispersion of the particles.

On the other hand, the mechanical vibrational responses of the rod were independently captured using a high-speed digital camera (Panasonic Lumix DMC-FZ200; image resolution: 1280 $\times$ 720 pixels; recording frequency: 100 fps) directed towards the rod’s free end to allow a front view of the motion. Simultaneously, a mirror was placed at 45 degree inclination to the side of the setup to allow the camera to also capture the side motion of the rod. Note that a transparent Perspex section with a matching diameter of the confining tube was used near the tip of the rod to allow optical access. Having obtained both the front and side views of the motion, the rod’s free end displacement in 3D could be reconstructed. An image processing algorithm was employed to calculate the centroid of the rod tip in each captured frame, which was then documented to create the displacement time series for both the front and side motions. The image processing and subsequent time series analysis were conducted using the open-source programme GNU Octave version 4.2.2 [20], utilising only the built-in functionalities offered by the ’image’ and ’signal’ packages. Further explanation of the cantilevered rod and the flow domain is provided in Sects. 3.7.1 and  3.7.2, respectively. These sections will cover the validation of the solid domain with free vibration and the validation of the fluid domain against the PIV data.

Different annulus Reynolds numbers (provided via Eq. 1) were achieved by changing the water flow rate. The annulus Reynolds number was determined by the following equation:

1 $$\begin{aligned} Re_{\text {ann}}= \frac{\rho _{\text {f}} d_{\text {h}} U_{\text {ann}}}{\mu _{\text {f}}} \end{aligned}$$

where $d_{\text {h}}$ is the hydraulic diameter, $U_{\text {ann}}$ is the flow velocity in the annulus, $\rho _{\text {f}}$ and $\mu _{\text {f}}$ are the density and dynamic viscosity of the fluid (water in our case).

A 60-s displacement time series was recorded for each test, from which the root mean square (RMS) displacement of the rod’s tip was calculated. Subsequently, a fast Fourier transform (FFT) of this time series was conducted to produce the power spectral density (PSD), uncovering the vibration modes. Measurements for different annulus Reynolds numbers were obtained sequentially as the Reynolds number increases from minimum to maximum; then, several additional measurements were repeated as the Reynolds number decreases when reducing the power of the pump. Note that the method for motion tracking adopted here builds upon the techniques developed by our group for FSI and axial-FIV studies, and the reader can refer to these techniques in [19, 21–25].

## Numerical Methodology

### Overview

The fluid and solid domains are solved separately and coupled using a two-way fluid–structure interaction (FSI) algorithm. In fact, this study adopts the finite volume method (FVM) for discretising both the solid and fluid domains to ensure consistency and clarity in FSI coupling, as well as to facilitate the future transition to a monolithic FSI solver. To streamline the implementation process, this study employs Foam-Extend version 4.0, a community-developed branch of the OpenFOAM software, supplemented with the solids4foam toolbox by Cardiff et al. [26].

Building upon and extending the numerical methodology outlined by Salachna et al. [14], this study aims to further explore and validate the applicability across a wider range of Reynolds numbers and flow configurations. The subsequent subsections will detail the governing equations and discretisation methods employed in this study.

### Solid Deformation

The governing equation for solid deformation is derived from the force equilibrium equation. The Hookean model, a linear stress model, incorporates the small-strain assumption, wherein the deformation is presumed to be small relative to the size of the body, and the second-order term in the finite strain tensor is neglected. This approach, while facilitates analysis, has limitations in capturing all deformations and can lead to errors, especially during rotations [27]. This is not of concern here, because rotations are not present. The equation for solid displacement is presented as follows:

2 $$\begin{aligned} \rho _s \frac{\partial ^2 u_i}{\partial t^2}+[\lambda + \mu ]\frac{\partial }{\partial x_i}\left( \frac{\partial u_j}{\partial x_j}\right) +\mu \frac{\partial ^2 u_i}{\partial x_j^2}+\rho _s f_i = 0 \end{aligned}$$

Here, *u* represents the solid displacement, $\rho _s$ is the solid’s density, $\mu$ and $\lambda$ are the Lame’s constants that represent the elasticity of the material, and *f* is the body force applied, specifically gravity in this case. Equation 2 is then rearranged to be solved with explicit treatment in the linear solver to enhance stability [28], as shown in Eq. 3.

3 $$\begin{aligned}  &   \rho _s \frac{\partial ^{2} u_i}{\partial t^{2}} -\underbrace{ [2 \mu + \lambda ]\frac{\partial }{\partial x_i} \left( \frac{\partial u_j }{\partial x_j} \right) }_{\text {Implicit}} \nonumber \\  &   \quad - \underbrace{\frac{\partial }{\partial x_i} \left( \mu \frac{\partial u_i}{\partial x_j} + \lambda \delta _{ij} \frac{\partial u_i}{\partial x_i} +[\mu +\lambda ] \frac{\partial u_j}{\partial x_j} \right) }_{\text {Explicit}} =-\rho _s f_i \end{aligned}$$

The FVM employed in this study stores information at the cell centroid, referred to as the cell-centred finite volume, a technique widely adopted in commercial CFD applications [29, 30]. This method necessitates cell-centre-to-vertex interpolation. In this study, the cell-centre-to-vertex interpolation employs a least-squares method, which estimates values at the vertex by optimally fitting a plane to adjacent cell centres. This approach is favoured over the commonly used inverse distance method for its superior accuracy, regardless of mesh quality [31].

The solid unsteady equation of motion from Eq. 3 in the x-direction, discretised using second-order temporal and spatial methods [32], is as follows:

4 $$\begin{aligned} a_P u_P^{[t]} - \sum _{i=N} a_i u_i^{[t]} = R_P \end{aligned}$$

where the subscripts *P* and *N* denote the owner and neighbouring cells, the superscript denotes the time step, $a_P$ and $a_i$ represent the diagonal and neighbouring coefficients, respectively, and $R_P$ is the source term; all defined as follows.

5 $$\begin{aligned} a_P= &   \frac{9 \rho _P V_P}{4 \Delta t^2} + \sum _{i=f} \left( 2 \mu _i + \lambda _i \right) \frac{S_i}{\Delta x_i} \end{aligned}$$

6 $$\begin{aligned} R_P= &   \rho _{P} V_P \left[ \frac{3 u_P^{[t-1]}}{\Delta t^2}- \frac{3 u_P^{[t-2]}}{4 \Delta t^2} + \frac{2}{\Delta t} \left( \frac{\partial u}{\partial t} \right) _P^{[t-1]}\right. \nonumber \\  &   \quad \left. - \frac{1}{2 \Delta t} \left( \frac{\partial u}{\partial t} \right) _P^{[t-2]} \right] \nonumber \\  &   - \sum _{i=f} \left( 2 \mu _{i} + \lambda _{i} \right) \frac{k_i \left( \frac{\partial u}{\partial x} \right) _i^{[t]} - k_P \left( \frac{\partial u}{\partial x} \right) _P^{[t]}}{{\Delta x_{i}}} S_i \nonumber \\  &   + \sum _{i=f} n_i q_i^{[t]} S_i + \sum _{i=f} \rho _P b_i^{[t]} V_p \end{aligned}$$

Here, the subscript *f* denotes the surrounding faces; *S* and *V* denote the surface area and cell volume, respectively; $k_f$ and $k_P$ are the non-orthogonal and skewness correction vectors, respectively; *q* is the explicitly treated term in the solid governing equation of motion (Eq. 3); and b is the source term.

A matrix of linear algebraic equations is formed by assembling it for N control volumes in the computational domain and applying the aforementioned discretisation methods. A segregated algorithm is employed, where the solution vectors for the x-, y-, and z- components are solved individually and then recoupled using an outer fixed-point iteration.

### Arbitrary Lagrangian–Eulerian (ALE) Formulated Fluid Mesh

The fluid flow is modelled based on the principles of mass and momentum conservation, adapted for a dynamic mesh through the arbitrary Lagrangian–Eulerian (ALE) formulation, which allows the fluid mesh to move independently from the fluid flow based on the boundary conditions. The ALE-modified Navier–Stokes equations are given as follows:

7 $$\begin{aligned} \frac{\partial U_i}{\partial t}+\frac{ \partial }{\partial x_j}(U_i - U_{w,i}) U_j=- \frac{1}{\rho _f} \frac{\partial P}{\partial x_i}+\nu \frac{\partial }{\partial x_j}\left( \frac{\partial U_i}{\partial x_j}\right) \nonumber \\ \end{aligned}$$

where *U* is the flow velocity, $\rho _f$ is the density of the fluid, and $U_w$ is the velocity of the mesh, solved using the Laplace equation as follows:

8 $$\begin{aligned} \frac{\partial }{\partial x_i} \left( \gamma \frac{\partial U_{w,i}}{\partial x_j} \right) = 0 \end{aligned}$$

Here, $\gamma$ is the diffusion coefficient, which governs the distribution of the fluid mesh upon motion, given as the quadratic of the inverse distance from the FSI interface for optimised preservation of mesh quality during fluid mesh deformation.

The fluid equations are discretised similarly to the solid equation, using second-order schemes for both temporal and spatial discretisation. A main focus of this investigation has been the discretisation of the convection terms in the momentum and turbulent transport equations. The simulation begins with the application of the first-order upwind scheme (FOUS) and progresses to higher-order schemes such as the second-order upwind scheme (SOUS), the central differencing scheme (CDS), and the Quadratic Upwind Interpolation for Convective Kinematics (QUICK) scheme [33], and the cubic interpolation scheme.

The pressure field is obtained by using the iterative Pressure Implicit with Splitting of Operators (PISO) algorithm [34] combined with the Rhie–Chow interpolation modified for moving mesh [32]. This study employs three PISO iterations that adjust the pressure field to achieve the desired velocity field, thus satisfying the mass continuity equation. Increasing the number of iterations has been shown to remove transient flow behaviour near the free end. Thus, while aiming to ensure numerical stability, an excessive number of iterations lead to overly smoothing out small-scale fluctuations and do not accurately reflect the desired transient dynamics of the actual flow.

Next, the Laplacian equation of motion in Eq. 8, formulated for the ALE dynamic mesh, is discretised using a second-order central difference scheme with non-orthogonal and skewness correction, similar to the explicitly treated terms in the solid discretised equation of motion in Eq. 6. Although this approach has disadvantages, such as instability and reduced accuracy, particularly in cases of large mesh distortion in flows through small gaps, it offers a straightforward and fast alternative.

### High-Reynolds-Number URANS Models

The range of annulus Reynolds numbers involved in this study falls within the turbulent flow region. The Reynolds averaged Navier–Stokes (RANS) approach was therefore employed. This approach decomposes the instantaneous velocity and pressure field into mean, *U*, and fluctuating, *u*, components and derives transport equations for the mean component. These transport equations for the Reynolds averaged velocity field, as shown in Eq. 9, include an additional set of terms corresponding to the time averaged product of the velocity fluctuations, which are called the Reynolds stresses; these represent the mixing effect of turbulence on momentum transport and require modelling.

9 $$\begin{aligned} \frac{\partial U_i}{\partial t}+\frac{ \partial }{\partial x_j}(U_i U_j)  &   =- \frac{1}{\rho _f} \frac{\partial P}{\partial x_i}\nonumber \\  &   \quad + \frac{\partial }{\partial x_j}\left( \nu \frac{\partial U_i}{\partial x_j}-\overline{u_i u_j}\right) +F_i \end{aligned}$$

The RANS equations, Eq. 9, are usually closed by one of two types of methods: firstly, the Eddy viscosity (EVM) approach, based on the Boussinesq hypothesis, which relates the Reynolds stresses to the turbulent viscosity and mean flow strain rate, and secondly, the Reynolds stress (RSM) approach, which directly solves transport equations for the individual components of the Reynolds stresses.

The EVM model tested here is the k-$\omega$ shear stress transport (SST) model, fully described in [35], while the RSM model tested is the Launder, Reece, and Rodi (LRR) model as proposed by Launder et al. [18].

Furthermore, in computationally intensive simulations with strong two-way FSI coupling, using a high-Reynolds-number turbulence model with wall functions for the modelling of the effects of near-wall turbulence significantly enhances efficiency and stability, by removing the need to employ meshes fine enough to resolve the near-wall viscous sublayer. The “universal” wall law, also referred to as the log-law, is used to calculate the wall shear stress (which is used as the wall boundary condition to the momentum equations) from the value of the velocity vector at the near-wall node [36].

#### Eddy Viscosity Model (EVM)

The Eddy viscosity model (EVM) follows the Boussinesq hypothesis, which assumes the Reynolds stresses as follows:

10 $$\begin{aligned} R_{ij}  &   =-\rho _{\text {f}} \overline{u_i u_j} = \mu _{\text {t}} \left( \frac{\partial U_i}{\partial x_j}+\frac{\partial U_j}{\partial x_i} \right) \nonumber \\  &   \quad - \frac{2}{3} \left( \rho _{\text {f}} k + \frac{\mu _t}{2} \frac{\partial U_k}{\partial x_k} \right) \delta _{ij} \end{aligned}$$

where $\mu _{\text {t}}$ is the turbulent viscosity and *k* is the turbulent kinetic energy. Here the turbulent viscosity is a function of the turbulent kinetic energy and in this case the parameter $\omega$ is equivalent to $\epsilon / k$. All types of effective viscosity models (EVM) involve a transport equation for the turbulent kinetic energy, *k*, and then depending on the type of EVM model, a second transport equation for either $\epsilon$ or $\omega$. The EVM model tested here is the k-$\omega$ Shear Stress Transport (SST) model, fully described in [35].

#### Reynolds Stress Model (RSM)

For the Reynolds stress model (RSM) closure, the six components of the Reynolds stresses are modelled through the solution of corresponding transport equations of the generic form given by Eq. 11.

11 $$\begin{aligned}  &   \rho _{\text {f}} \frac{\partial \overline{u_i u_j}}{\partial t} + \rho _{\text {f}} U_k \frac{\partial \overline{u_i u_j}}{\partial x_k} = \rho _{\text {f}} P_{ij} - \rho _{\text {f}} \epsilon _{ij} + \rho _{\text {f}} \phi \nonumber \\  &   \quad + \frac{\partial }{\partial x_k} \left[ (\mu +\mu _\text {t}) \frac{\partial \overline{u_i u_j}}{\partial x_k} \right] \end{aligned}$$

The terms on the left-hand side and the generation rate term, $P_{ij}$, given below, are exact.

12 $$\begin{aligned} P_{ij}=- \left( \overline{u_j u_k} \frac{\partial U_i}{x_k} + \overline{u_j u_k} \frac{\partial U_j}{x_k} \right) \end{aligned}$$

The remaining terms in Eq. 11 are modelled as proposed by Launder et al. [18] in what has become known as the Launder, Reece, and Rodi (LRR) model. For effective implementation of the LRR model, an initial estimate of the Reynolds stresses within the flow domain is first obtained. This is achieved by first determining the turbulent dissipation rate, $\epsilon$ field, using the standard k-$\epsilon$ model [37]. Subsequently, the Reynolds stresses ($R_{ij}$) are computed by applying the Boussinesq approximation, as delineated in Eq. 10.

### Modelling of Near-Wall Turbulence

As noted previously, using high-Reynolds-number turbulence models with wall functions for the modelling of the effects of near-wall turbulence significantly enhances efficiency and stability, by removing the need to employ meshes fine enough to resolve the near-wall viscous sublayer. It has been observed in similar FIV simulations involving vibrating rods with both ends fixed [38] that varying the number of radial mesh elements and comparing low- and high-Reynolds-number models, the latter with wall functions, result in negligible differences in the predictions [9].

As explained in detail in a number of publications, among them [39], in the wall function approach, the near-wall node is placed far enough away from the wall to be outside the wall viscous sublayer, and then, the “universal” wall law, also referred to as the log-law, is used to calculate the wall shear stress (which is used as the wall boundary condition to the momentum equations) from the value of the velocity vector at the near-wall node. Further details can be found in reference [39].

### Fluid–Structure Interaction

At the FSI interface, the kinematic and dynamic boundary conditions are applied as follows, respectively:

13 $$\begin{aligned} u_{F,i}= &   u_{S,i}. \end{aligned}$$

14 $$\begin{aligned} n_i \sigma _{F,i}= &   n_i \sigma _{S,i}. \end{aligned}$$

Here, the subscripts *F* and *S* refer to the fluid and solid domains, respectively; subscript *i* is the cell quantity at the FSI interface, while $n \sigma$ represents the normal stresses. The fluid displacement, $u_F$, here refers to the displacement of the fluid mesh.

Initially, a solution of the flow transport equations in the fluid domain provides the force distribution over the solid surface. A subsequent solution of the elastic deformation over the solid domain provides the deformation of the solid boundaries, caused by the fluid motion. The solid boundary deformation is used to generate a mesh for the deformed fluid domain. The flow field is then updated by solving the flow transport equations over the new mesh. The updated flow field subsequently provides a new force field over the solid surface. The above steps are repeated until the solid domain shows no further changes from one iteration to the next.

A detailed explanation of the generalised grid interpolation (GGI) used for the non-conformal mesh at the FSI interface, along with the FSI coupling decomposition and the FSI coupling algorithm, is provided in Appendices A.1, A.2, and A.3.

### Computational Domains and Mesh Details

#### Improved Single-Material Solid Domain Assumption

To simplify and accelerate the simulation, the lead shot-filled cantilevered rod (Fig. 1) is assumed to be made of a single material, i.e. an empty rod whose mechanical properties have been determined to reproduce the mechanical response of the actual lead shot-filled rod. The rod vibration frequency as predicted according to the Euler–Bernoulli equation in quiescent water, given in Eq. 15, together with the added mass expression for confined vibrating rods in Eq. 16 [40], was used to predict modes of vibration and estimate the properties of the single-material model.

15 $$\begin{aligned} f_1= &   \frac{{\beta _1}^2}{2 \pi } \sqrt{\frac{EI}{({\dot{m}}_{\text {rod}}+{\dot{m}}_{\text {add}})L_{\text {rod}}^4}} \end{aligned}$$

16 $$\begin{aligned} {\dot{m}}_{\text {add}}= &   \rho _f \frac{\pi }{4} d_{\text {ro}}^2 \frac{1+(d_{\text {ro}}/d_{\text {ti}})^2}{1-(d_{\text {ro}}/d_{\text {ti}})^2} \end{aligned}$$

Here, $\beta$ is the influence factor, given as 1.875 for the first mode of vibration of a cantilever [41]. $L_{\text {rod}}$, $d_{\text {ro}}$, $d_{\text {ti}}$ are the geometrical properties defined in Fig. 1; ${\dot{m}}_{\text {add}}$ is the linear added mass evaluated using Eq. 16; ${\dot{m}}_{\text {rod}}$ is the linear mass density of the rod measured experimentally; $\rho _{\text {f}}$ is the density of the fluid in the confining tube; and *EI* is the flexural rigidity of the rod.

The lead shots inside the rod do not contribute to the flexural rigidity of the rod [16, 17, 19]. Hence, we assume the flexural rigidity is solely from the steel cladding and consider the linear mass density of the lead-filled rod while using the Euler-Bernoulli equation (Eq. 15) to predict the first mode of vibration.

Furthermore, if we compare the single-material model featuring solid cylinder geometry against the hollow cylinder geometry, similar to the thickness of the steel cladding, then both demonstrate a similar first mode of vibration. However, the hollow cylinder geometry achieves significantly greater computational efficiency [15]. Therefore, considering these factors, the geometry of the single-material rod is chosen to be similar to that of the steel cladding. A detailed comparison between the properties of the rod in the experiment and the model is given in Table 1.

**Table 1:** Properties of the cantilever rod in the experiments and the single-material model

Properties	Experiment	Hollow cylinder model
Young’s modulus, $E_{\text {steel}}$ [GPa]	193	202.26
Rod density, $\rho _{\text {steel}}$ [kg/$m^3$]	7990	33,676.3
Cap density, $\rho _{\text {aluminium}}$ [kg/$m^3$]	2740	33,676.3
Cap mass, ${\dot{m}}_{\text {aluminium}}$ [g]	2.1	2.1
Cap length, $L_{\text {cap}}$ [mm]	2.0	1.32
Rod fillings	Lead shot	Empty
Rod linear mass density, ${\dot{m}}_{\text {rod}}$ [kg/m]	0.588	0.588
Second moment of inertia, I [$\hbox {mm}^4$]	194.4	194.4
First mode of vibration in water, $f_1$ [Hz]	3.7	3.7

**Fig. 2:**
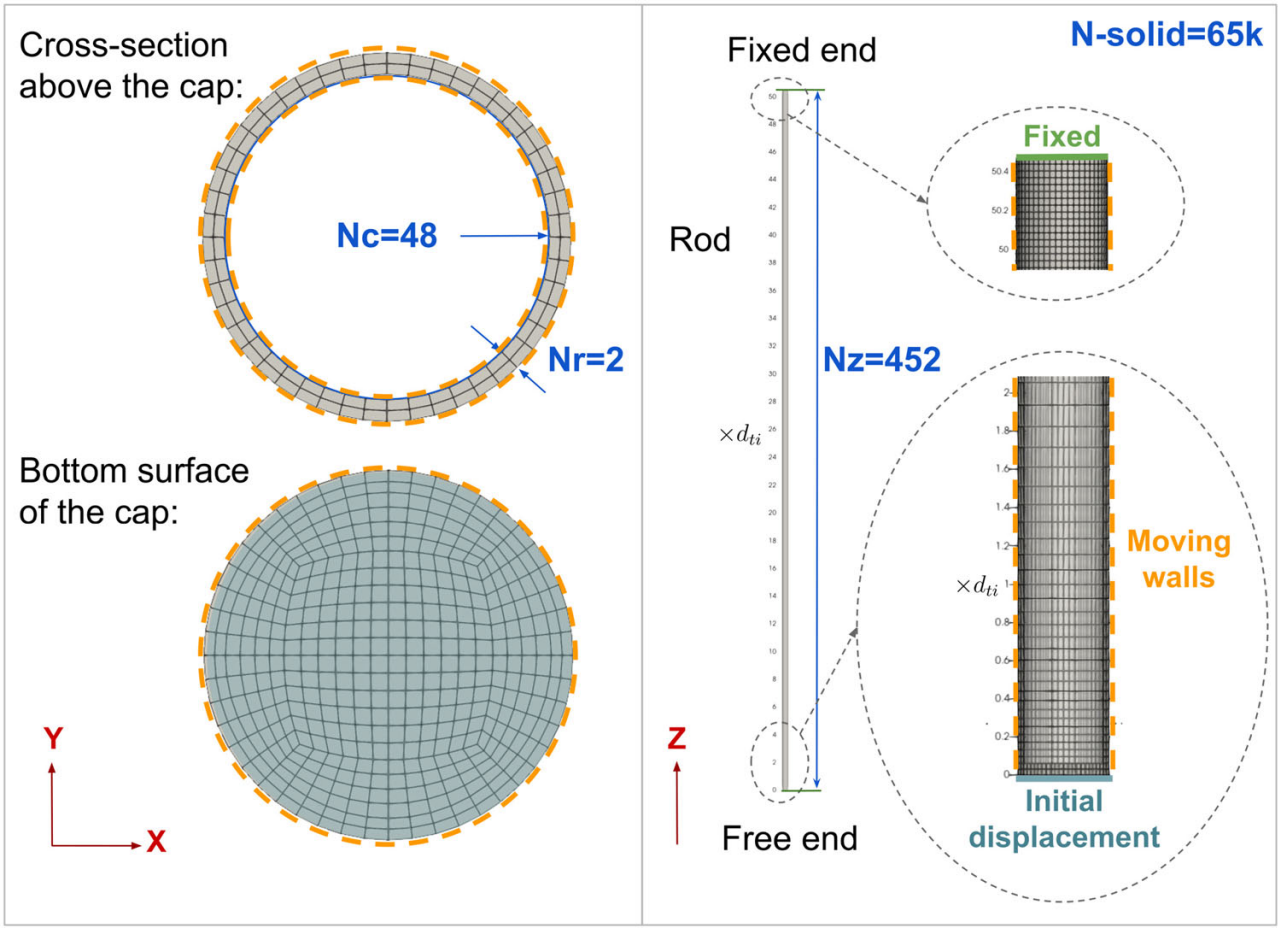
Mesh view of the hollow cylinder model and its boundary conditions

**Fig. 3:**
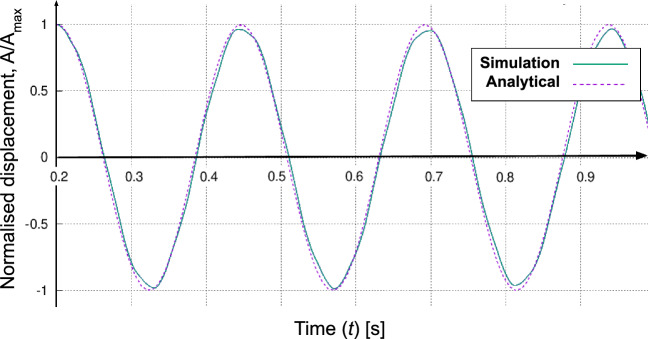
Normalised displacement time series from the simulation and the Euler–Bernoulli equation for the free vibration case

Figure 2 displays the mesh for the solid domain, formed using a structured hexahedral mesh and the O-grid method for the cylinder. To ensure accuracy, a free vibration test of the solid domain was conducted, and two aspects were considered: the frequency of vibration from the Euler–Bernoulli equation with zero added mass and the preservation of amplitude at every vibration as shown in Fig. 3. Subsequently, a mesh independence analysis was conducted. The desired mesh requires a finer axial mesh near the fixed end, while the width, Nr, achieved acceptable accuracy with just two intervals. In fact, Fig. 2 presents the optimised solid mesh for this study, refined to enable self-excited axial-FIV simulation for replicating unsteady flow behaviour near the free end and enhancing cylinder fidelity for a more accurate circular circumference representation.

#### Fluid Domain

**Fig. 4:**
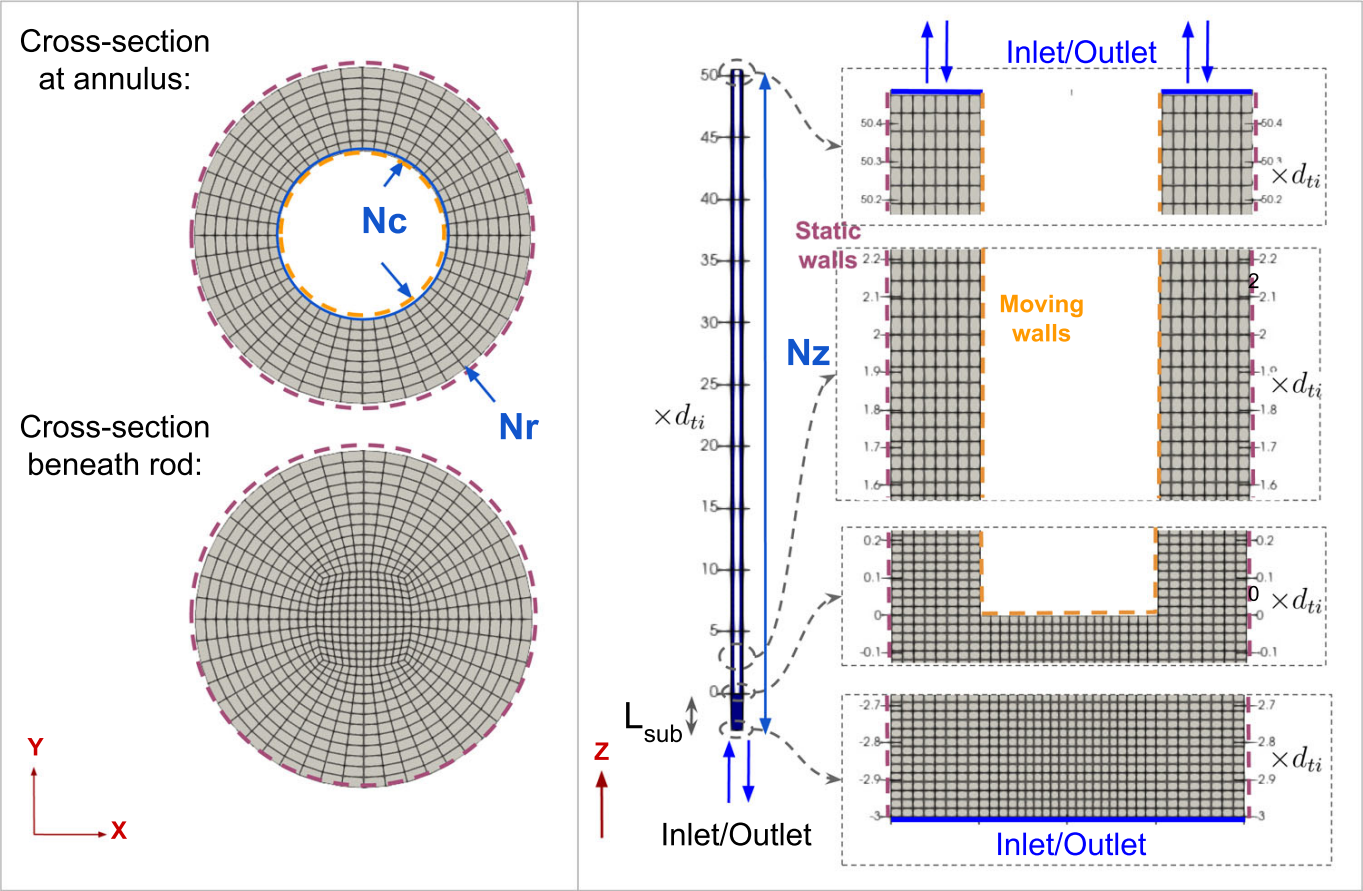
Mesh views and boundary conditions of the fluid domain

The geometry of the flow domain is depicted in Fig. 4, and its simplifications at the inlet are described in Sect. 3.5. The fluid properties were sourced from the NIST Reference Fluid Thermodynamic and Transport Properties Database [42] using fluid density and viscosity corresponding to the average experimental temperature of 295 K.

Similar to the solid mesh, the fluid mesh given in Fig. 4 consists of a structured hexahedral mesh and an O-grid for the cylinder, with the surface at the bottom of the rod being conformal with the solid domain. For both flow configurations, a finer mesh circumferentially (Nc), axially (Nz), and radially (Nr) than the mesh shown in Fig. 4 yielded consistent results, despite the $y^*_{\text {min}}$ falling to around 20, which is below the recommended near-wall distance for the high-Reynolds-number wall function. The mesh descriptions from performing mesh independence analysis are given in Table 2.

**Table 2:** Mesh description for the fluid mesh for both flow configurations

Variables	Free–fixed	Fixed–free
Number of axial intervals, Nz	1218	1470
Number of radial intervals across annulus, Nr	8	12
Number of circumferential intervals, Nc	48	48
Length below rod’s free end, $L_{\text {sub}}$ [$\times d_{\text {ti}}]$	3	9
Number of fluid control volumes, N-fluid	620k	1100k
Minimum dimensionless distance of near-wall nodes from rod, $y^*_{\text {min,rod}}$	24–29	$\sim$30
Minimum dimensionless distance of near-wall nodes from tube, $y^*_{\text {min,tube}}$	36–37	$\sim$41

Furthermore, Table 2 shows slight differences between the two configurations. Firstly, the number of radial intervals in the annulus was adjusted to achieve better resolution with appropriate first near-wall node distance for the high-Reynolds-number wall function. Secondly, the flow channel beneath the rod was extended from 3 to 9 times the tube diameter until the velocity profile across the xy-plane no longer changed in the axial direction.

### Initial and Boundary Conditions

The solid domain, depicted in Fig. 2, boundary conditions are set for two variables: displacement and traction force. These are applied to three types of surfaces. The first is the fixed end at the top surface of the cantilever. The second, the initial displacement surface located at the bottom free end, is where initial displacement is enhanced by applying a traction force parallel to the surface. This force is applied for a duration equal to the dominant frequency of vibration, thereby eliminating inertial motion upon the removal of the traction force for the free vibration case. Lastly, there are the moving walls, which comprise all remaining surfaces, both internal and external to the rod. In the case of self-excited FIV, the bottom surface, previously the initial displacement surface, is also set as moving walls.

The boundary conditions for the fluid domain, shown in Fig. 4, encompass four types of surfaces. The inlet surface is positioned three times the tube diameter below the rod’s free end for the free–fixed configuration and at the annulus surface adjacent to the fixed end for the fixed–free configuration. A preliminary computation of fully developed pipe flow, with a mesh that mirrors that of the inlet boundary, was conducted, to provide fully developed flow inlet conditions for the free–fixed (upward) flow configuration. At the outlet plane zero gradient boundary conditions are employed. For all walls, no slip boundary conditions are applied, and, as already noted, the value of the wall shear stress is related to that of the velocity vector at the near-wall node, through the wall function approach.

## Results and Discussions

### Overview

As explained previously, our goal is to advance the existing numerical methodology for axial-FIV [14] by extending its applicability to a wider range of Reynolds numbers and flow configurations. This endeavour has encountered two primary challenges. Firstly, simulations at higher Reynolds numbers demand significantly more computational resources, especially for the two-way fluid–structure interaction (FSI), where initial attempts revealed that larger Reynolds numbers necessitate longer computational time for the solid solver to achieve convergence. Consequently, further optimisation of the solid domain was undertaken. Secondly, the capacity to accurately simulate transient flow behaviour necessitated reassessment in the context of changing flow configurations.

### Simulation of Transient Flow Behaviour

**Fig. 5:**
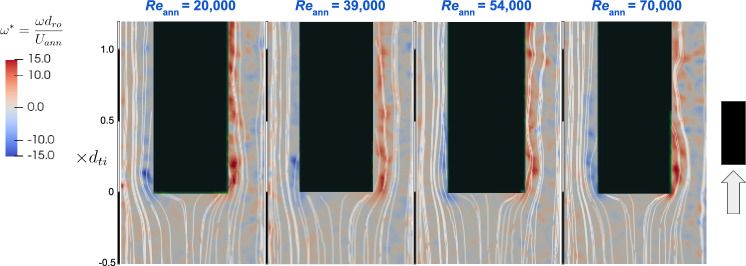
Contour plots of instantaneous normalised vorticity, $\omega d_{\text {ro}}/U_{\text {ann}}$, obtained from experiments, for different annulus Reynolds numbers for the free–fixed configuration

**Fig. 6:**
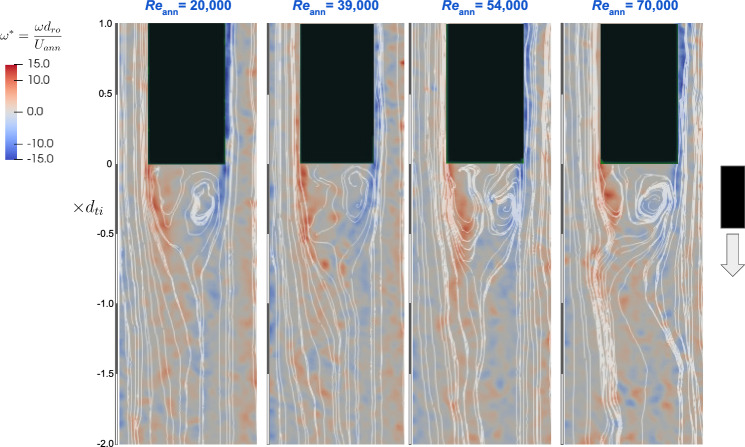
Contour plots of instantaneous normalised vorticity, $\omega d_{\text {ro}}/U_{\text {ann}}$, obtained from experiments, for different annulus Reynolds numbers for the fixed–free configuration

To provide insights into the underlying flow dynamics, the flow fields measured from the experimental PIV setup at distinct Reynolds numbers are presented in Figs. 5 and 6. The instantaneous vorticity contours, normalised as $\omega d_{\text {ro}}/U_{\text {ann}}$, highlight regions of heightened turbulent vorticity along the rod’s sides and near the bottom surface edges. Notably, despite the increase in Reynolds number, the dimensionless vorticity levels and the contour patterns remain relatively consistent.

In the free–fixed configuration (Fig. 5), the flow demonstrates a stagnation region at the rod tip end with separation bubbles generated at the 90 degree corners of the rod end and carried by the flow along the rod sides. In contrast, the fixed–free configuration (Fig. 6) distinctly showcases flow separation, where vortices are shed from the free end’s edges, generating an unsteady wake downstream of the free tip end. This vortex shedding extends roughly one tube diameter downstream and is consistent across the range of Reynolds numbers investigated.

An essential aspect of achieving accurate RMS vibration amplitude is the employment of a physical fluid model capable of reproducing the oscillatory flow behaviour near the rod’s free end, which drives the rod oscillations. Prior to this, the flow domain is validated at a mid-value annulus Reynolds number of 35.1k, by comparing the mean velocity profiles at several key axial positions along the flow channel against PIV measurements, which will be presented later in this section.

To induce self-excited FIV, URANS models should reproduce velocity and pressure fluctuations of turbulent flow, maintaining the alternating lift coefficient on the rod’s surface to enhance vibration amplitude. For numerical stability, turbulent variables are discretised using the stable first-order upwind scheme (FOUS). As noted previously, the discretisation of convection of momentum is tested with several schemes, namely second-order upwind, central differencing, quadratic upstream interpolation scheme (QUICK), and cubic interpolation discretisation.

**Fig. 7:**
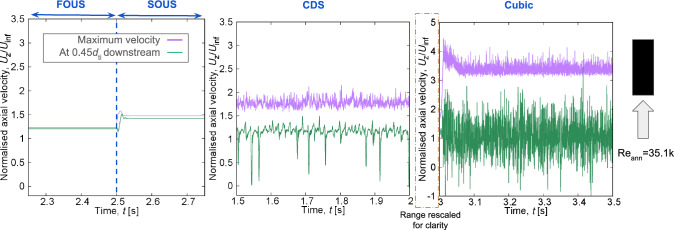
Axial flow velocity time series for different convection schemes for the momentum equations, denoted as FOUS (first-order upwind scheme), SOUS (second-order upwind scheme), CDS (central differencing scheme), and cubic (cubic interpolation scheme)

**Fig. 8:**
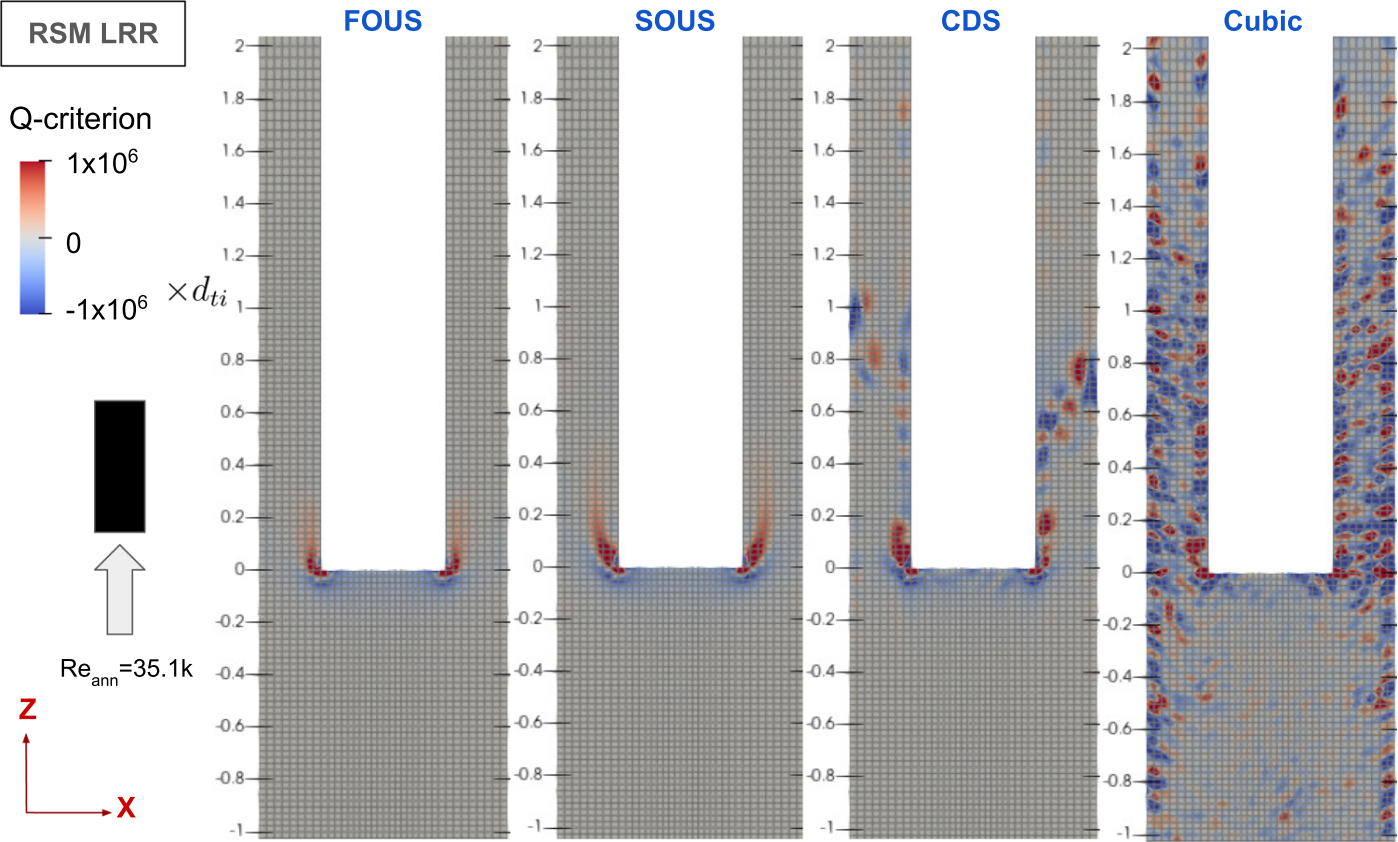
Contour plots of instantaneous Q-criterion for varying convection schemes for the momentum equations for the free–fixed configuration at the validation Reynolds number

Figures 7 and 8 show that both first-order upwind (FOUS) and second-order upwind (SOUS) schemes result in steady flow predictions. In contrast, the second-order central difference (CDS) and third-order cubic interpolation (Cubic) schemes are capable of reproducing flow instabilities, including vortices shed at the free end’s edge. Thus, it is concluded that the central difference and cubic interpolation schemes are more suitable for discretising the convective transport of momentum.

**Fig. 9:**
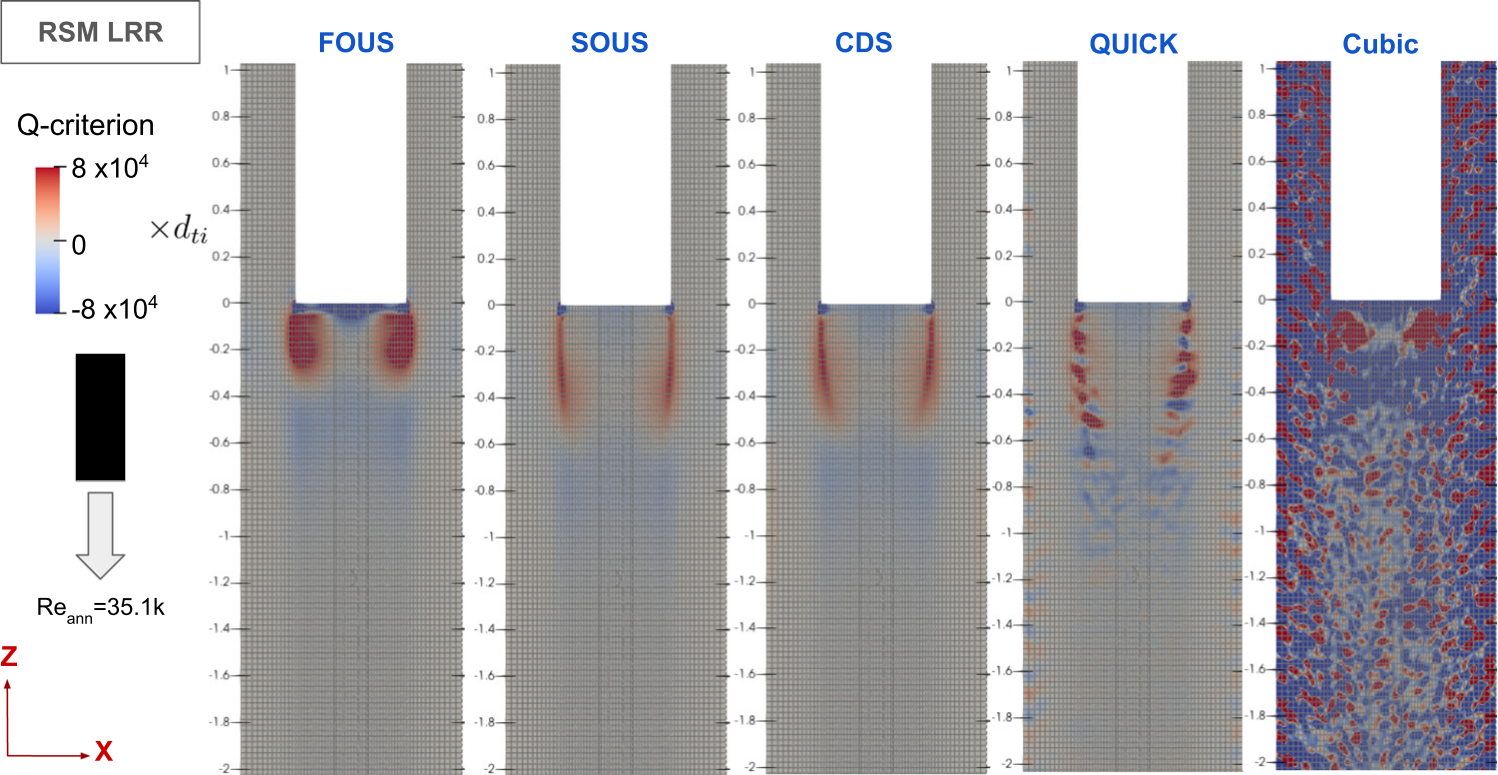
Contour plots of instantaneous Q-criterion for varying convection schemes for the momentum equations for the fixed–free configuration at the validation Reynolds number

For the fixed–free configuration, reproducing the unsteady flow behaviour near the free end with the same numerical schemes as in the free–fixed configuration proved more challenging. Comparing the effects of the convection schemes applied to the momentum equation, Fig. 9 displays Q-criterion contour plots depicting vortices shed from the rod’s free end. As the order of the discretisation scheme increases, there is noticeable increase in the length and complexity of the shed vortices. Only the cubic interpolation scheme, however, returns a substantial number of vortices both upstream and downstream of the rod’s free end. Upon applying two-way FSI coupling, however, the flow returns to steady-state behaviour, suggesting that there is a problem with the cubic interpolation’s implementation in OpenFOAM.

**Fig. 10:**
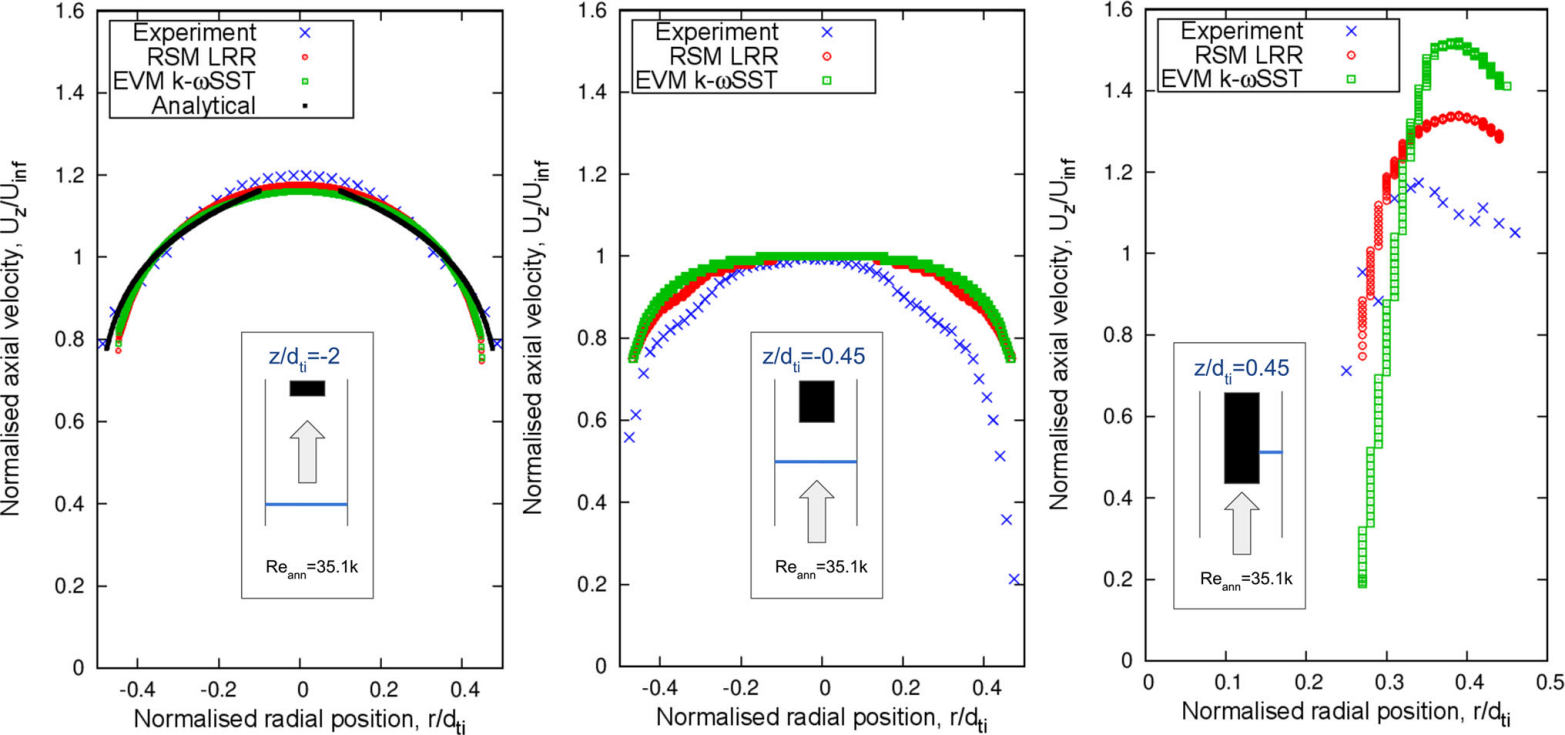
Average velocity profiles at: (left) 2$d_{\text {ti}}$ upstream, (middle) 0.45$d_{\text {ti}}$ upstream, and (right) 0.45$d_{\text {ti}}$ downstream the rod’s free end from the PIV data of Cioncolini et al. [16] against the simulations with different URANS models at the validation annulus Reynolds number of 35.1k

Attention is now turned to comparisons between predicted and measured profiles of the mean velocity, presented in Fig. 10, for the selected validation Reynolds number case of 35.1k.

At the location of 2 diameters upstream (below) of the free end, the PIV data show that the approaching flow is symmetric and fully developed. Agreement between the profiles predicted by both models and the one measured using PIV is very close. Closer to the free end, just approximately half a diameter below, the measurements indicate that the velocity profile becomes flatter. That said, the axial velocity profile produced by the stress transport model (LRR) is still in close agreement with the measured variation, while the one resulting from the use of the k-$\omega$ SST model appears to show a slower development from the fully developed to the flatter state. At a location about half a diameter downstream (above) the free end, the measurements show that across the right side of the annulus the flow accelerates. This trend is reproduced by both models, but they both overestimate the acceleration, with the k-$\omega$ SST returning the faster, and hence more erroneous velocity.

The possible reasons for these predictive deviations are the lack of complete symmetry in the experimental flow field and also the possible lack of geometric symmetry, with the rod not being located at exactly the centre of the tube. In regions where the flow undergoes strong acceleration, like the rod tip, the effect of even minor non-symmetries can be amplified.

### Comparison of Predictions Against Experiments

#### Free–Fixed Configuration

**Fig. 11:**
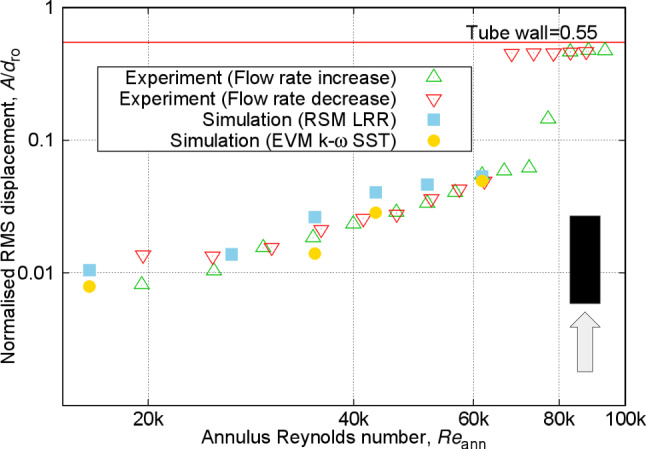
Normalised RMS amplitude of vibration (observed displacement divided by rod diameter) at varying annulus Reynolds numbers for the free–fixed configuration

Moving on to FIV aspects, Fig. 11 demonstrates the variation in the RMS amplitude of vibration for the free–fixed configuration. The displacement time series measured at the tip of the rod initially exhibits small-amplitude random fuzzy type-1 motion at the lower range of Reynolds numbers measured in this study. As the flow velocity increases, the RMS amplitude of vibration rises till it reaches a critical point where instability emerges. In cantilevered rods, this instability manifests as flutter-like motion, alternating between large deflection near the surface of the tube channel and small-amplitude vibrations [17, 43]. Further increase in flow velocity causes the rod to deflect to one side with small-amplitude random vibration while remaining in the deflected position. Note that similar dynamic motions have been observed in various configurations, encompassing both cross- and axial flows, for both high-stiffness and flexible rods, and in systems where the rod is either clamped at both ends or configured as a cantilever [17, 43, 44].

The amplitudes of vibration presented in Fig. 11 are the resultant radial displacements in polar coordinates for annulus Reynolds numbers ranging between approximately 30k and 70k. In this range, the displacements measured from both the front and side planes were relatively similar. However, significant differences emerged outside this Reynolds number range. This is because, at the relatively low Reynolds numbers, small amplitudes are easily influenced by far-field sources of vibrations, such as the pump [45] and the flow loop configuration [46].

On the other hand, at the higher end of Reynolds numbers, possible imperfections in the experimental setup and the rod’s structure could have amplified the differences in displacements. Furthermore, comparing the measurements from the increasing and decreasing flow rates in Fig. 11 reveals notable differences in the critical Reynolds number for the onset of instability. For increasing rates, the transition to unstable motions with relatively large amplitudes (above 0.1$d_{\text {ro}}$) appears at a Reynolds number around 75k, whereas for the decreasing rates, this transition occurs at around 65k. This indicates hysteresis in the free–fixed configuration, an observation typically found for similar setups [17, 43].

**Fig. 12:**
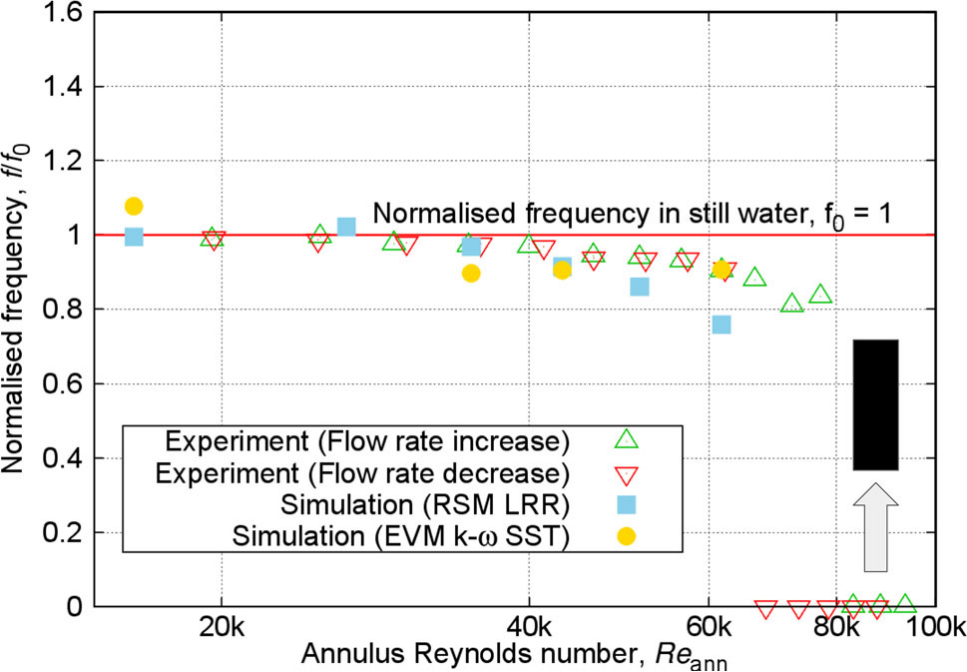
Normalised frequency of vibration (observed frequency divided by frequency of vibration in still water, equal to 3.72 Hz) at varying annulus Reynolds numbers for free–fixed configuration

The dominant frequency of vibration was extracted from the power spectral density (PSD) by performing a fast Fourier transform (FFT) on the displacement time series for experimental measurements at increasing and decreasing flow rates, as well as for both URANS models (see Appendix B). Figure 12 presents an average value of the frequency values measured from both front and side planes. Note that the frequencies in both planes were nearly identical. This implies that far-field excitation sources and imperfections in the rod’s structure minimally affect the vibration frequency, in contrast to the vibration amplitude. Moreover, at higher Reynolds numbers, a null frequency can be observed, signifying the deflection region where the rod begins to contact the tube surface, thus eliminating the identification of the dominant frequency.

#### Fixed–Free Configuration

**Fig. 13:**
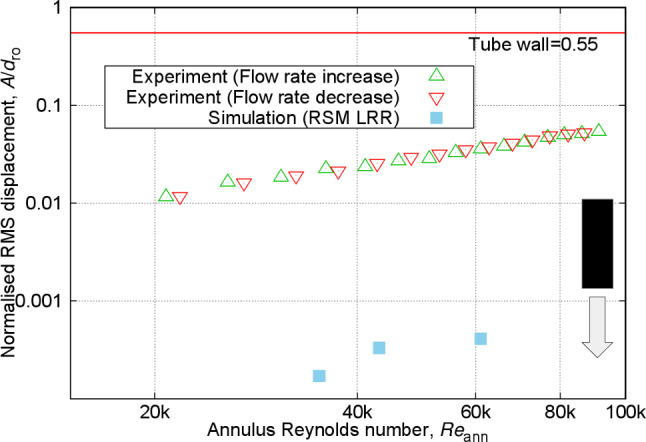
Normalised RMS amplitude of vibration at varying annulus Reynolds numbers for the fixed–free configuration

Figure 13 shows the RMS displacement amplitudes against the flow Reynolds number for the fixed–free configuration. The figure demonstrates nearly similar vibration amplitudes measured in the experiments for both increasing and decreasing flow rates across all Reynolds numbers, indicating that the fixed–free configuration consistently exhibits small-amplitude, random fuzzy period-1 type of motion. As previously discussed for the free–fixed configuration, the blunt-end shape distinctly enhances the stability of the vibrational motion at higher Reynolds numbers, contrasting with the flutter-like instability observed with curved ends in the fixed–free configuration [17]. The predicted amplitudes of vibration are considerably lower than the observed values.

**Fig. 14:**
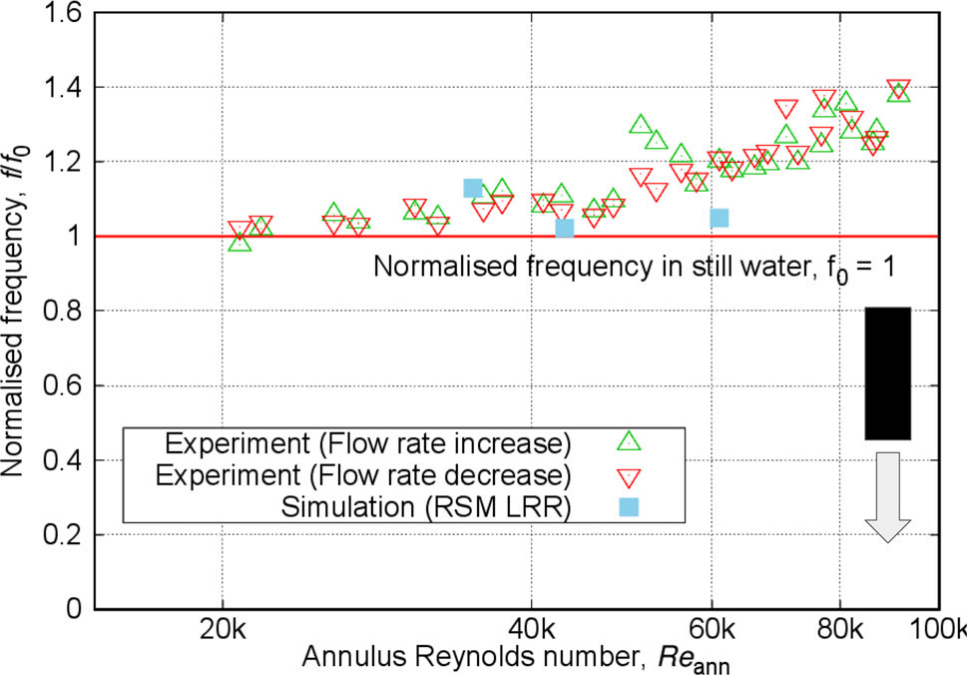
Normalised frequency of vibration at varying annulus Reynolds numbers for the fixed–free configuration

The frequency of vibration measured experimentally for the fixed–free configuration, shown in Fig. 14, reveals that, with increasing the annulus Reynolds numbers, a gradual rise from the value of the dominant frequency of vibration in still water is evident. This trend opposes that of the free–fixed configuration shown previously in Fig. 12. That said, these results are consistent with previous experimental results reported for rods with semi-spherical curved tip end [17].

### Discussions

For the free–fixed configuration, within the range of annulus Reynolds numbers below flutter instability, for both URANS models, the predictions align well with the experimental results for both RMS amplitude and dominant frequency of vibration shown in Figs. 11 and 12. The LRR model shows better accuracy than the k-$\omega$ SST model when compared to the PIV measurement of average velocity profile near the free end as depicted in Fig. 10. The larger overprediction of the downstream reattachment length from the k-$\omega$ SST model as compared to the LRR model as discussed in Sect. 4.2 is related to the overestimation of the generation rate of turbulence in complex flows, which in turn leads them to predict lower flow instabilities and a lower RMS amplitude of vibration.

Next, for the frequency of vibration in Fig. 12, the LRR model indicates a gradual decrease in frequency values from the dominant frequency of vibration in still water with an increase in annulus Reynolds number, reflecting the same trend as that observed in the experimental measurements. Meanwhile, the k-$\omega$ SST model exhibits minor scatter in the results, specifically at Reynolds numbers of 35.1k and 43.1k, where it displays slightly lower frequencies compared to the highest Reynolds number of 61.7k. Despite these discrepancies, both simulations fall within an acceptable range when considering the experimental uncertainty and the shorter time series durations used in the FFT analysis for the simulations as compared to the experiment.

Moreover, comparing the stability and computational efficiency of the two URANS models, the LRR model has been found to remain stable for the Reynolds number values tested, apart from the highest one (61.7k). At this point, the combination of large pressure force on the FSI surface and the large oscillation amplitude causes large deformations of the mesh, compromising simulation stability and causing divergence. The k-$\omega$ SST model, on the other hand, exhibited numerical stability problems at all Reynolds numbers.

For the fixed–free configuration, only the LRR model is presented since the k-$\omega$ SST model showed a very small, non-physical vibrational response and did not produce a self-excited FIV (described in Appendix B). Figure 13 shows that increasing the annulus Reynolds numbers to 61.7k consistently raises the RMS amplitude of vibration from the simulation, though it remains two orders of magnitude lower than that of the experimental measurements. This indicates that without accurately reproducing the oscillatory flow behaviour and the associated fluid forces near the free end, a sufficient RMS amplitude of vibration is unattainable. The rise in RMS amplitude here is directly linked with the increase in freestream flow velocity.

The corresponding frequency of vibration in Fig. 14 closely matches the experimental results, registering just slightly above the dominant frequency of vibration in still water. This near alignment reflects the limited impact of flow on the vibration frequency, further emphasising its dependence on the rod’s properties [9–15].

**Fig. 15:**
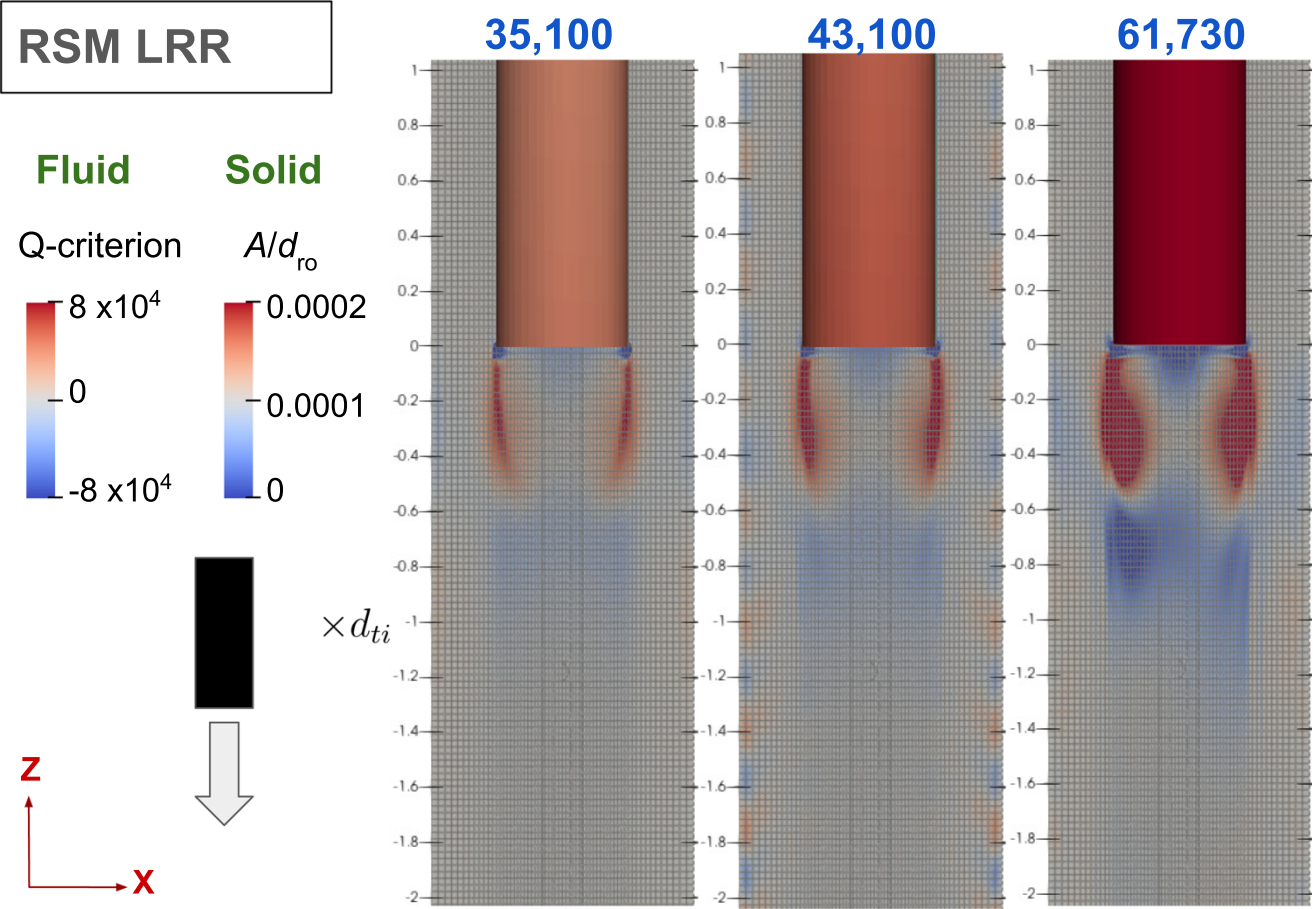
Contour plots of instantaneous Q-criterion at different annular Reynolds numbers using RSM LRR model for the fixed–free configuration

The instantaneous Q-criterion contour plots for the RSM LRR model in Fig. 15 reveal a contrast to the experimental PIV measurements presented in the previous section (Fig. 5). Across all Reynolds numbers, the experiments display asymmetry, whereas the RSM LRR model shows symmetry on both sides of the tube’s centreline at the annulus upstream of the rod’s free end. Although the increase in Reynolds number did not alter the length of flow separation, as corroborated by the computed Q-criterion contours in Fig. 15, it did intensify the vorticity in the downstream flow separation region.

The paper identifies potential mechanisms reflected in the simulation outcomes. It is suggested that the boundary layer on the downstream surface of the blunt end is effectively shielded from the upstream freestream flow by a large flow separation region downstream, as shown in Fig. 15. This condition likely results in the simulation predicting steady-state behaviour, consequently reducing the fluid forces exerted near the free end.

In contrast, the free–fixed configuration, illustrated in Fig. 8 (using central difference scheme (CDS), similar to Fig. 15’s fixed–free configuration), experiences direct impacts from the freestream flow on the downstream flow separation. This direct interaction facilitates the URANS turbulence modelling approach in capturing crucial interaction between the freestream flow and flow separation, resulting in vortices shedded from the edge and reproducing the desired transient flow behaviour near the free end.

## Final Remarks

This comprehensive study on axial-FIV on blunt-end cantilevers provides improved understanding of the complexities of flow behaviours and their impact on nuclear fuel bundles. By systematically increasing/decreasing the annulus Reynolds numbers while considering both fixed–free and free–fixed configurations, and analysing the corresponding vibration responses, this work has exposed how the Reynolds number and the axial flow direction influence the stability and amplitude of the flow-induced vibrations of the problem in hand. Employing computationally efficient URANS turbulence models, including the RSM LRR and EVM k-$\omega$ SST, alongside strong two-way FSI coupling, the study significantly highlights the importance of representing transient flow behaviour near the free end of the vibrating rod for the accurate prediction of axial-FIV. In fact, this work exposed the influence of convection schemes on capturing these transient behaviours, providing valuable guidance for future simulations and model improvements.

It should be noted that the implications of this research extend far beyond academic interest, directly impacting the design and safety of nuclear reactors. Understanding the differences in vibrational response with varying Reynolds numbers, between different axial flow directions, as well as between blunt and curved-ended rods, aids in better prediction of potential FIV issues, thereby contributing to the development of safer and more efficient nuclear power plants.

## Figures and Tables

**Fig. 16:**
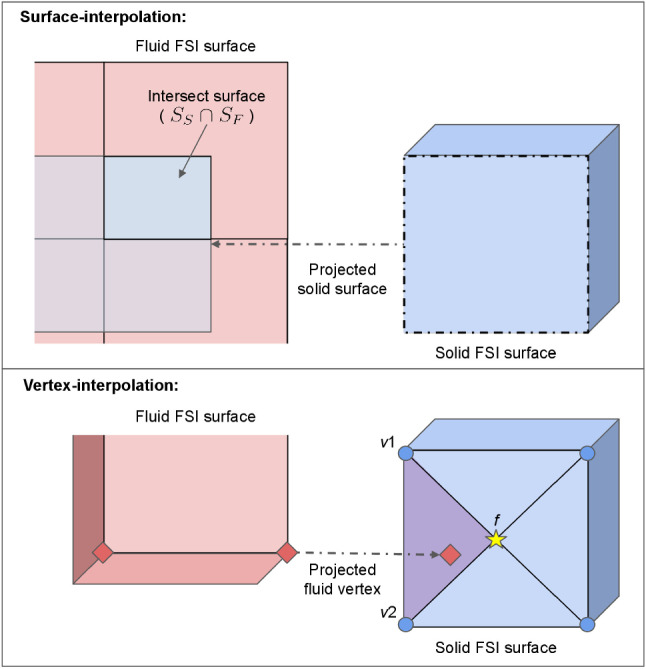
Illustration of the FSI interface interpolation

**Fig. 17:**
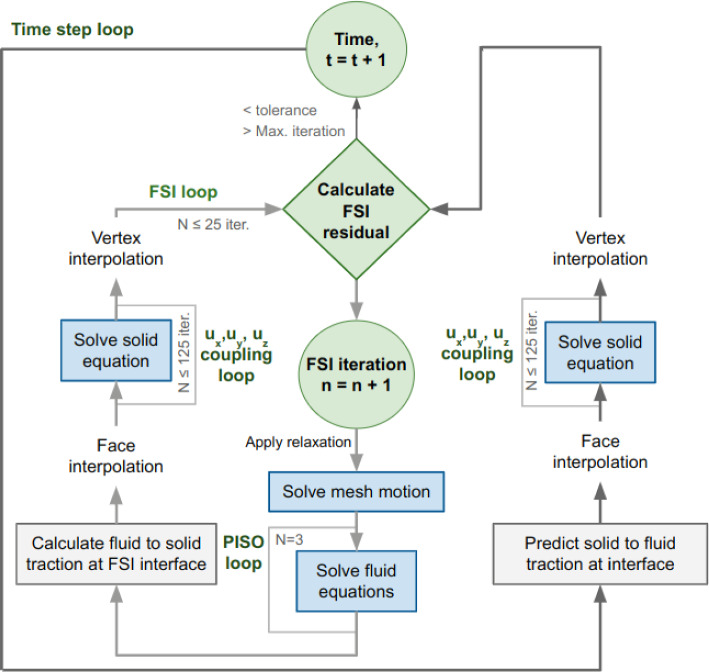
Flowchart for FSI coupling algorithm

**Fig. 18:**
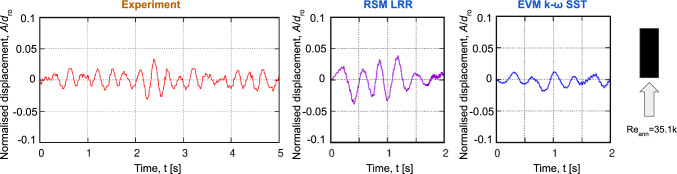
Normalised displacement time series for the experiment and the simulations for the free–fixed configuration

**Fig. 19:**
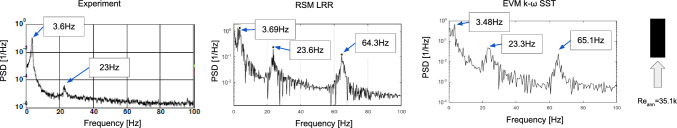
Power spectral density of displacement time series from the experiment and the simulations for the free–fixed configuration

**Fig. 20:**
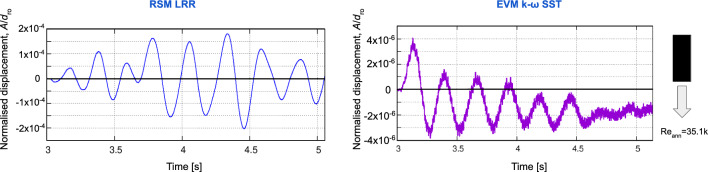
Normalised displacement time series for RSM LRR model (left) and EVM k-$\omega$ SST (right) for the fixed–free configuration

## Fluid–Structure Interaction (FSI)

### FSI Interpolation

At the FSI interface, where the fluid and solid meshes are non-conformal, interpolations are performed as shown in Fig. 16. The traction force from the fluid flow, located at the cell faces at the interface, requires face-interpolation. In contrast, the transfer of displacement from the vertices of the solid mesh at the interface necessitates vertex interpolation. Face interpolation is executed using the Generalised Grid Interface (GGI) interpolation method, as proposed by Beaudoin and Jasak [47]. This method involves summing the weighted contributions from the opposing fluid surfaces that intersect the solid surface, as follows:

A1 $$\begin{aligned} t_{S,j}= &   \sum _k w_{j,k} \ t_{F,k}. \end{aligned}$$

A2 $$\begin{aligned} w_{j, k}= &   \frac{S_{S, j} \cap S_{F, k}}{S_{S, j}}. \end{aligned}$$

where subscripts *j* and *k* represent the face centres at the interface for solid (*S*) and fluid (*F*) mesh, respectively.

For vertex interpolation, the solid side of the interface is first decomposed into a triangle formed by a face centre and two neighbouring vertices. Then, the vertex from the fluid cells at the interface is projected onto this triangle, and linear interpolation using the triangle’s vertices’ known displacements determines the displacement at the projected fluid vertex’s location as follows:

A3 $$\begin{aligned} u_F = \left[ w_1 * u_{S(f)} \right] + \left[ w_2 * u_{S(v1)} \right] + \left[ w_3 * u_{S(v2)} \right] . \end{aligned}$$

Here, the subscripts *f*, $v_1$, and $v_2$ refer to the vertices of the projected fluid vertex on the solid, which comprises a face centre and two vertices. The interpolation weightage, *w*, is determined based on the position of the projected fluid vertex.

### Dirichlet–Neumann (DN) Decomposition

Two-way FSI coupling was achieved using the Dirichlet–Neumann (DN) procedure. Initially, the Dirichlet boundary condition, given in Eq. 13, establishes vertex interpolation, Eq. A3 for the displacement of the solid mesh at the fluid mesh vertices, enabling the solution of the fluid flow equation across the entire fluid domain. Subsequently, the Neumann boundary condition, given in Eq. 14, applies face interpolation, Eqs. A1 and A2, allowing the fluid flow equation to provide traction information at the cell centre of the boundary cells. This traction, encompassing pressure and stresses at the boundary faces, is used to determine stresses within the solid mesh’s cell centre, aiding in solving the solid displacement equation for the entire solid domain. In comparison with other FSI boundary condition methods like Neumann–Dirichlet (ND) and Robin–Dirichlet (RN), the DN decomposition has shown greater robustness and computational efficiency, particularly in dynamic mesh [48]. Figure 17 illustrates the flow chart of this FSI coupling algorithm.

In a strong two-way FSI coupling, the FSI residual, ${\textbf{r}}$, calculated as the difference between the displacements of the solid and fluid meshes at the FSI interface, as given in Eq. A4, is solved using an iterative FSI coupling algorithm to ensure stable and efficient convergence.

A4 $$\begin{aligned} {\textbf{r}}_{i}^{k} = {\textbf{u}}_{s,i}^{k} - {\textbf{u}}_{f,i}^{k} \end{aligned}$$

As shown in Fig. 17, each time step requires performing multiple FSI iterations, which can be computationally exhaustive, considering the existing iterations performed on the solid and fluid domain. A quasi-Newton method, which approximates the inverse of the Jacobian of the residual matrix (IQN-ILS), as developed by Degroote [48] and detailed in Appendix A.3, was used. This method has proved to be more efficient and stable compared to the commonly used Gauss–Seidel (GS) algorithm and has also been used for relatively similar simulations involving high-stiffness materials in turbulent flow [12, 13, 49].

### IQN-ILS Coupling Algorithm

The interface quasi-Newton method which approximates the inverse least-squares of the Jacobian (IQN-ILS) fluid–structure interaction (FSI) coupling algorithm by Degroote [48] is described as follows. To calculate the residual between fluid and solid domain at the interface (Eq. A4), the displacement of the fluid domain is approximated as follows:

A5 $$\begin{aligned} {\textbf{u}}_{f,i}^{k+1}&= {\textbf{u}}_{f,i}^{k}+ \Delta {\textbf{u}}_{f,i}^{k} \nonumber \\&= {\textbf{u}}_{f,i}^{k}+\widehat{\left( \frac{1}{{\textbf{R}}_{i}^{'k}}\right) }\left( \Delta {\textbf{r}}_i^k \right) \end{aligned}$$

Here, the subscripts *i* and *f* refer to the vertices at the FSI interface and the fluid domain, respectively, while the superscript *k* denotes the FSI iteration and $\mathbf {R'}$ represents the residual matrix. The desired residual in the FSI coupling is zero; hence, the difference between the desired and the current residual, $\mathbf {r_k}$, is written as follows.

A6 $$\begin{aligned} \Delta {\textbf{r}}_i^k&= {\textbf{0}} - {\textbf{r}}_i^k \nonumber \\&=- {\textbf{r}}_i^k \end{aligned}$$

To approximate the inverse of the Jacobian, vectors are constructed by leveraging information from the preceding FSI iteration. Therefore, in the initial two FSI coupling iteration ($k=$0, 1), the displacement and subsequent residual are calculated similar to the GS algorithm as follows:

A7 $$\begin{aligned} {\textbf{u}}_{f,i}^{k+1} = {\textbf{u}}_{f,i}^{k} + \omega ^{k} {\textbf{r}}_{i}^{k} \end{aligned}$$

The superscripts *k* and *k*+1 refer to the previous and current FSI coupling iteration, and $\omega$ is the relaxation factor. For strong FSI coupling in incompressible flow, a very low relaxation is required to ensure stability and avoid divergence, especially at the initial few FSI coupling iterations [50, 51]; hence, after careful optimisation, the relaxation is set to 0.05.

Next, the difference in the solid displacement and residual between the current, *k* and previous, *k*-1, iteration is calculated as follows, respectively.

A8 $$\begin{aligned} \Delta {\textbf{r}}_{i}^{k-1}= &   {\textbf{r}}_{i}^{k} - {\textbf{r}}_{i}^{k-1} \end{aligned}$$

A9 $$\begin{aligned} \Delta {\textbf{u}}_{s,i}^{k-1}= &   {\textbf{u}}_{s,i}^{k} - {\textbf{u}}_{s,i}^{k-1} \end{aligned}$$

The calculated solid displacement at the vertices in Eq. A9 is not the final solid displacement used for the new time step, but rather the FSI coupling based on the DN procedure. Next, sets of differences in Eqs. A8 and A9 are stored as columns in matrices, $\mathbf {\underline{V}}$ and $\mathbf {\underline{W}}$, respectively, while the rows correspond to the vertex stencil, *i*, at the FSI interface. A row in the matrices is presented as follows.

A10 $$\begin{aligned} \mathbf {\underline{V}}^k= &   \begin{bmatrix} \Delta {\textbf{r}}_i^{k-1}&\Delta {\textbf{r}}_i^{k-2}&\cdots&\Delta {\textbf{r}}_i^{1}&\Delta {\textbf{r}}_i^{0} \end{bmatrix} \end{aligned}$$

A11 $$\begin{aligned} \mathbf {\underline{W}}^k= &   \begin{bmatrix} \Delta {\textbf{u}}_{s,i}^{k-1}&\Delta {\textbf{u}}_{s,i}^{k-2}&\cdots&\Delta {\textbf{u}}_{s,i}^{1}&\Delta {\textbf{u}}_{s,i}^{0} \end{bmatrix} \end{aligned}$$

To accelerate convergence, the use of similar information, such as residuals and solid displacements from previous time steps, *t*-*q*, has been proven to reduce the number of time steps required to reach convergence [48]. A modification that incorporates the reuse of information from the previous iteration into the ${\textbf{V}}$ and ${\textbf{W}}$ matrices is presented as follows.

A12 $$\begin{aligned} {\textbf{V}}^k= &   \begin{bmatrix} \mathbf {\underline{V}}^{k}&{\textbf{V}}^{n}&\cdots&{\textbf{V}}^{n-q+2}&{\textbf{V}}^{n-q+1} \end{bmatrix} \end{aligned}$$

A13 $$\begin{aligned} {\textbf{W}}^k= &   \begin{bmatrix} \mathbf {\underline{W}}^{k}&{\textbf{W}}^{n}&\cdots&{\textbf{W}}^{n-q+2}&{\textbf{W}}^{n-q+1} \end{bmatrix} \end{aligned}$$

A vector is approximated as a linear combination of known residual changes, $\Delta {\textbf{r}}_i$, given as follows:

A14 $$\begin{aligned} \Delta {\textbf{r}}_i \approx {\textbf{V}}^k {\textbf{c}}^k \end{aligned}$$

where ${\textbf{c}}^k$ is the coefficient of the decomposition, a matrix with a single row and a similar number of columns as matrices ${\textbf{V}}$ and ${\textbf{W}}$. The Householder transformation is used to decompose the matrix ${\textbf{V}}$ into an orthogonal matrix ${\textbf{Q}}$ and an upper triangular matrix ${\textbf{R}}$ [52], given as follows.

A15 $$\begin{aligned} {\textbf{V}}^k = {\textbf{Q}}^k {\textbf{R}}^k \end{aligned}$$

The change between subsequent displacements, $\Delta {\textbf{u}}^s$, is obtained by first calculating the coefficient vector ${\textbf{c}}^k$ through back substitution of a triangular matrix, and subsequently using a linear combination, as follows, respectively.

A16 $$\begin{aligned} {\textbf{R}}^k {\textbf{c}}^k= &   {\textbf{Q}}^{kT} \Delta {\textbf{r}} \end{aligned}$$

A17 $$\begin{aligned} \Delta {\textbf{u}}_s= &   {\textbf{W}}^k {\textbf{c}}^k \end{aligned}$$

From the definition of the FSI residual in Eq. A4, the change in residual between subsequent FSI iterations is given as follows.

A18 $$\begin{aligned} \Delta {\textbf{r}} = \Delta {\textbf{u}}_s - \Delta {\textbf{u}}_f \end{aligned}$$

Hence, combining Eqs. A17 and A18 gives the change in the fluid displacement, $\Delta {\textbf{u}}_f$, as follows.

A19 $$\begin{aligned} \Delta {\textbf{u}}_f = {\textbf{W}}^k {\textbf{c}}^k - \Delta {\textbf{r}} \end{aligned}$$

The product of the approximation of the inverse Jacobian and the change in residual from the quasi-Newton iteration from Eq. A5 is solved using the least-square transformation in Eq. A19 and the approximation of the changes in the residual in Eq. A6, to give the following relation.

A20 $$\begin{aligned} \widehat{\left( \frac{1}{{\textbf{R}}_{i}^{'k}}\right) }\left( \Delta r_i^k \right) = {\textbf{W}}^{k} {\textbf{c}}^{k} + {\textbf{r}}_{i}^{k} \end{aligned}$$

Finally, the displacement of the fluid vertices is updated for the new FSI coupling iteration, $u_{f,k+1}$ by inserting Eq. A20 into the quasi-Newton iteration in Eq. A5, and subsequently, the new residual, $r_{k+1}$, is calculated and compared against the convergence criteria.

A21 $$\begin{aligned} {\textbf{u}}_{f,i}^{k+1}= &   {\textbf{u}}_{f,i}^{k} + {\textbf{W}}^{k} {\textbf{c}}^{k} + {\textbf{r}}_i^{k} \end{aligned}$$

A22 $$\begin{aligned} {\textbf{r}}_{i}^{k+1}= &   {\textbf{u}}_{s,i}^{k+1} - {\textbf{u}}_{fi}^{k+1} \end{aligned}$$

If the residual is below the convergence criteria, the IQN-ILS coupling procedure is repeated for the new iteration (*k*=*k*+1) until the residual is reached and continues to the new time step (*t*=*t*+1).

## Validating Self-Excite Axial-FIV

For the validation of the self-excited FIV, fluid and solid meshes described in Sects. 3.7.1 and 4.2 were used. To ensure stability, a one-way FSI coupling was performed for one second, followed by the two-way FSI. The normalised displacement time series are shown below.

Figure 18 demonstrates that for both URANS models, the root mean square (RMS) amplitude of displacements achieved good agreement within the experiment’s uncertainties. Fast Fourier transforms (FFTs) were performed on the displacement time series for the simulations above, while the experiments were extracted from a full 300 s displacement time series, to yield the power spectral densities (PSD) as follows.

Figure 19 indicates good agreement within the uncertainties of the experiments for both the first and second vibration modes using both URANS models. However, the simulations reveal an additional peak at a higher frequency, absent in the experimental data, where a power spectral density (PSD) with a moving-average filter is used on the experimental data to eliminate higher frequency noises from distant vibration sources. Both the displacement time series and the first modes of vibration do not show significant differences between the x- and y-directions; hence, subsequent results present the resultant displacement and average modes of vibration.

Using similar numerical schemes as in the free–fixed configuration, the normalised displacement time series for the fixed–free configuration in Fig. 20 reveals that the RSM LRR model managed to produce a self-excited FIV, but with a magnitude of RMS amplitude of vibration two orders lower than the experimental measurement. Meanwhile, using the EVM k-$\omega$ SST did not produce a self-excited FIV, appearing non-physical and mainly due to numerical effects, similar to the results reported in published works [13, 14]. Hence, the mean RMS amplitude could not be extracted.

The FFT analysis performed on the displacement time series for the RSM LRR model resulted in an average first mode of frequency equal to 4.2 Hz, very close to the 4.1 Hz obtained from the experiment. The lesson learned here is the importance of reproducing transient flow behaviour near the free end to accurately reproduce the RMS amplitude of vibration, while the first modes of vibration, which mostly rely on the solid model, can be reproduced even with a small amplitude of vibration.
